# Delivering safer conception services to HIV serodiscordant couples in Kenya: perspectives from healthcare providers and HIV serodiscordant couples

**DOI:** 10.7448/IAS.20.2.21309

**Published:** 2017-03-08

**Authors:** Kenneth Ngure, Grace Kimemia, Kristin Dew, Njambi Njuguna, Nelly Mugo, Connie Celum, Jared M Baeten, Renee Heffron

**Affiliations:** ^a^ Department of Public Health, Jomo Kenyatta University of Agriculture and Technology, Nairobi, Kenya; ^b^ Department of Behavioral Sciences, Partners in Research Health and Development, Thika, Kenya; ^c^ Department of Human Centered Design and Engineering, University of Washington, Seattle, WA, USA; ^d^ Department of Clinical Sciences, Partners in Research Health and Development, Thika, Kenya; ^e^ Center for Clinical Research, Kenya Medical Research Institute, Kenya; ^f^ Department of Global Health, University of Washington, Seattle, WA, USA; ^g^ Department of Epidemiology, University of Washington, Seattle, WA, USA; ^h^ Department of Medicine, University of Washington, Seattle, WA, USA

**Keywords:** HIV prevention, Kenya, pregnancy, safer conception, serodiscordant couples

## Abstract

**Introduction**: For HIV serodiscordant couples in resource-limited settings, pregnancy is common despite the risk of sexual and/or perinatal HIV transmission. Some safer conception strategies to reduce HIV transmission during pregnancy attempts are available but often not used for reasons including knowledge, accessibility, preference and others. We sought to understand Kenyan health providers’ and HIV serodiscordant couples’ perspectives and experiences with safer conception.

**Methods**: Between August 2015 and March 2016, we conducted key informant interviews (KIIs) with health providers from public and private HIV care and fertility clinics and in-depth interviews (IDIs) and focus group discussions (FGDs) with HIV serodiscordant couples participating in an open-label study of integrated pre-exposure prophylaxis (PrEP) and antiretroviral therapy (ART) for HIV prevention (the Partners Demonstration Project). An inductive analytic approach identified a number of themes related to experiences with and perceptions of safer conception strategies.

**Results**: We conducted 20 KIIs with health providers, and 21 IDIs and 4 FGDs with HIV serodiscordant couples. HIV clinic providers frequently discussed timed condomless sex and antiretroviral medications while providers at private fertility care centres were more comfortable recommending medically assisted reproduction. Couples experienced with ART and PrEP reported that they were comfortable using these strategies to reduce HIV risk when attempting pregnancy. Timed condomless sex in conjunction with ART and PrEP was a preferred strategy, often owing to them being available for free in public and research clinics, as well as most widely known; however, couples often held inaccurate knowledge of how to identify days with peak fertility in the upcoming menstrual cycle.

**Conclusions**: Antiretroviral-based HIV prevention is acceptable and accessible to meet the growing demand for safer conception services in Kenya, since medically assisted interventions are currently cost prohibitive. Cross-disciplinary training for health providers would expand confidence in all prevention options and foster the tailoring of counselling to couples’ preferences.

## Introduction

The greatest burden of the HIV epidemic lies in sub-Saharan Africa, where a substantial proportion of infections occur in long-term HIV serodiscordant partnerships, that is where one partner is HIV infected and the other uninfected [1]. Despite the risk for sexual and perinatal HIV transmission facing HIV serodiscordant couples, couples report maintaining their fertility goals and experience pregnancy rates similar to those of the general population [2].

**Table 1.** Participant characteristics

Type of interview	In-depth interviews	Focus group discussions
*Members of HIV serodiscordant couples*		
N	42	25 (total participants – 4FGDs)
Age, Median (IQR)	31 (28–38)	32(28–35)
% Female	21 (50%)	14 (56%)
% HIV positive	21 (50%)	20 (80%)
Children, Median (IQR)	1 (1–2)	1 (0–1)
*Providers (participating in key informant interviews)*		
N	20	
Gynaecologist	11 (55%)	
Fertility clinics	5	
Jointly public and private clinics	4	
Safer conception research sites	2	
Nurse counsellors	5 (25%)	
Safer conception research sites	3	
Public health hospitals	2	
Clinical Officers	2 (10%)	
Safer conception research sites	1	
Public health hospitals	1	
Counsellors	2 (10%)	

There is a critical need to deliver an acceptable and scalable package of HIV risk reduction strategies for couples attempting pregnancy in low-resource settings which has untapped potential to impact the course of the HIV epidemic. An ideal package should combine efficacious HIV risk reduction strategies and encourage couples to select the methods they most prefer. Potential risk reduction strategies include antiretroviral therapy (ART) use by HIV-infected partners to suppress HIV viral levels, pre-exposure prophylaxis (PrEP) use by HIV-uninfected partners, condom use except during days with peak fertility, vaginal self-insemination when the female is the HIV-infected partner, medically assisted reproduction, screening and treatment of sexually transmitted infections, voluntary medical male circumcision when the male is the HIV-uninfected partner, and fertility screening to prevent unnecessary HIV exposure during pregnancy attempts in the face of infertility [3]. Current efforts to integrate HIV and reproductive health services provide opportunities to identify HIV serodiscordant couples with fertility intentions and promote safer conception when pregnancy is desired [15].

Pilot studies in Europe have demonstrated that safer conception interventions for HIV serodiscordant couples can help them achieve pregnancies without HIV transmission [10–12,16]. Interventions to reduce HIV risk during peri-conception periods are being piloted in Uganda and South Africa [17,18]. In Kenya, formative data on tool development and community perceptions are available [19] and HIV prevention services are expanding rapidly to include early initiation of ART and PrEP for people with substantial HIV risk, including HIV serodiscordant couples, enabling safer conception delivery to potentially include a menu of options that extends beyond the existing options of timed condomless sex and self-insemination [20]. In order to inform the design of a comprehensive safer conception intervention in Kenya, we explored HIV and reproductive health providers’ experiences counselling on safer conception and HIV serodiscordant couples’ preferences about safer conception strategies.

## Methods

### Study design

Between August 2015 and March 2016, we conducted in depth interviews (IDIs) and focus group discussions (FGDs) with heterosexual HIV serodiscordant couples and key informant interviews (KIIs) with health providers from public and private health settings.

### Study population

Couples were participants at the Thika, Kenya site of the Partners Demonstration Project, an open-label delivery study of integrated PrEP and ART among high-risk HIV serodiscordant couples in Kenya and Uganda. The couples were ≥18 years of age, sexually active, intending to remain as a couple for at least one year and were identified as being at higher risk for HIV-1 infection (expected incidence >5% per year without PrEP or ART) using a validated, empiric risk scoring tool. Recruitment strategies included conducting community outreach and liaising with local clinics to promote couples-based HIV testing [21]. Couples for this nested qualitative study were identified based on their responses to routinely administered surveys about fertility goals. Couples who indicated that they were currently trying or intended to start trying to become pregnant were invited to participate and scheduled individually for IDIs or FGDs at a separate time of their convenience. The criteria were the same for inclusion in FGD and IDI.

Additionally, the Partners Demonstration Project had a network of referral health providers, including obstetrician/gynaecologists nurse counsellors, clinical officers, and counsellors working in safer conception research clinics providing PrEP and ART free of charge, fertility centres (private specialist clinics that serve couples with fertility challenges and provide assisted reproduction interventions at a cost), and public hospitals with experience working with HIV serodiscordant couples desiring pregnancy and most are experienced in providing HIV care and treatment free of charge. These providers were approached to participate in KIIs.

### Data collection

Semi-structured guides were used to conduct KIIs, IDIs and FGDs by experienced social scientists who were not known to study participants. The duration for KIIs and IDIs was 30–60 minutes while FGDs were 45–90 minutes. Each session was conducted in English, Kiswahili or Kikuyu, according to the participant’s preference and consensus in the FGDs. KIIs focused on knowledge, attitudes and practices regarding safer conception practices. In-depth interviews (IDIs) focused on individual experiences being a member of an HIV serodiscordant couple with pregnancy goals and how these influenced perceptions of and barriers to reducing HIV risk during pregnancy attempts. Focus group sessions aimed to elicit community perceptions of safer conception strategies and opinions of the feasibility of different components that could be included in safer conception interventions. Each KII, IDI and FGD was recorded, transcribed, and translated into English. One KII declined to be recorded and hand written notes were used in place of a full transcript.

### Data analysis

Coding was conducted using a combination of an inductive approach based on the grounded theory [22] and a deductive approach [24]. Two coders (GT and KD) met to review categories and agree on a codebook [25] that was applied to all the transcripts thematically using Dedoose Version 7.0.23 software – 42% of all transcripts were double coded. The lead authors (KN and RH) reviewed all the emergent themes with the coders to ensure accuracy and consistency of interpretation. This multiple coding allowed the qualitative analogue of inter-rater reliability [26,27].

### Ethical approval

We obtained ethical approval for the study from the Kenya Medical Research Institute Ethical Review Committee and the University of Washington Institutional Review Board. All participants provided written informed consent.

## Results

### Participants

We conducted KIIs with 20 health providers including, 11 obstetricians and gynaecologists, 2 clinical officers, 5 nurse counsellors and 2 counsellors. All except the 5 obstetrician/gynaecologists were primary HIV service providers. With members of HIV serodiscordant couples, we conducted 4 FGDs (two FGDs with 14 HIV-infected women, one FGD with 6 HIV-infected men, and one FGD with 5 HIV-uninfected men) and 21 IDIs (12 couples with HIV-infected women and 9 with HIV-uninfected women). Members of HIV serodiscordant couples had a median age of 32 (IQR 28–37), 50% were women, 63% were HIV infected, and most had at least one child (Table 1).

We present results based on the safer conception strategies reported by HIV serodiscordant couples and healthcare providers under the following broad themes (a) antiretroviral-based methods (b) identifying the peak fertility period and timed conception (c) medically assisted reproduction (d) self-insemination and (e) counselling approach.

## Antiretroviral-based methods

Members of HIV serodiscordant couples frequently talked about PrEP and ART as prevention strategies that they would be willing to use to reduce peri-conception HIV risk. Many felt that these strategies were acceptable and sufficient for prevention. Couples reported that having sex and using ART and PrEP was the “easier” way to conceive (relative to self-insemination or other methods) and reported knowing other couples who have safely used these methods to conceive. Couples felt they had HIV protection from ART and PrEP even when condoms were not used. In spite of this, some participants reported that they could not accept the “risk” with antiretroviral-based safer conception, citing concerns about their partner’s adherence to either ART or PrEP.

*“Because like I am HIV positive and my husband is negative and he takes Truvada and I take ARV and you can see his health is good. It is hard for me to infect him and we are still okay and also my health continues to be okay so I think ARVs they help me; they are even my best friend…*” (HIV-infected females, FGD)

*“So it is advisable if you can use…the one who is not infected to use Truvada and the other one to use ARVs so that you reduce the chances of this one, who is infected to infect the other one if they are trying to get a child.”* (HIV-uninfected males, FGD)

Providers reported frequently discussing antiretroviral-based strategies (ART and PrEP) and many stressed the importance of ART. A number of providers talked about PrEP but they were not yet routinely recommending it since it has limited availability outside of research settings. These providers were keen to have PrEP delivered widely so that they could add it to the safer conception strategies they were offering couples. Providers at fertility clinics often lacked information about the prevention benefits of antiretrovirals and reported reluctance to recommend these methods. The gynaecologists and fertility specialists discussed antiretrovirals for post-exposure prophylaxis (PEP) but rarely as PrEP. Most providers talked about combining strategies, such as PrEP combined with ART or ART combined with sperm washing/intrauterine insemination (IUI).

*“I have seen the strategy of using drugs, that is ART to the positive partner and even PrEP to the negative partner, work. Then now you counsel them about fertility period so that they know how they can go about condomless sex during the period that the peak fertility is high*.” (Health provider, safer conception research site, KII)

*“Well I am not very aware of the new PEP and PrEP studies;……I think the safest way is really if it is the man it is the sperm wash*.” (Health provider, Private fertility clinic, KII)

## Identifying peak fertility and timed conception

Although couples reported high acceptability of timed condomless sex, many displayed limited knowledge of peak fertility days and how to identify them. Some providers corroborated this unfamiliarity among their clients, especially clients with limited education and familiarity with calendars. For these couples, providers reported that frequent reminders were important as well as working with the couple to track their fertility cycles when they returned for their follow-up visits. Providers felt challenged to find a medium for reminding couples about their fertile days. Most of the couples perceived conception to be a “chance” occurrence and that most couples have to attempt a few times to see success. However, some men expressed belief that if the fertile days were correctly determined, a pregnancy was sure to result and some HIV-infected men were concerned about the risk of HIV transmission during timed conception.

*“I would have become (pregnant) because now if you finish your periods like today, you go (have sex) tomorrow, you would get (pregnant)…because after my periods, the first day is over, the second and third one (gesturing 3 with her fingers)…some conceive one day before periods if you go (have sex)*.” (HIV-uninfected female, age 51)

*“We can have sex without a condom and then maybe by chance a child is conceived, by chance I also don’t get the disease.”* (HIV-uninfected female, IDI, age 33)

*“… [Timed condomless sex] is okay except now you never know, the woman can get, she can also be infected, now that is what we are afraid of again.”* (HIV-infected male, age 43)

Timed condomless sex was an acceptable strategy to most providers working in non-fertility clinics and many expressed the opinion that it has a very low risk of HIV transmission when practiced with ART use. In contrast, some providers especially those from providers in fertility clinics expressed opinions that timed condomless sex should not be recommended because there is a non-zero transmission risk. Providers also reported that couples used timed intercourse as single risk reduction strategy even when not on antiretroviral-based methods

*“So, they are not willing to go all that, so you find most of them on their own they have chosen a timed intercourse. It is the easier option, of course…”* (Health provider, Fertility clinic-public and private, KII).

## Medically assisted reproduction

Most couples were aware of some forms of medically assisted reproduction, especially sperm washing, but most lacked specific details of how the different techniques are done and which is appropriate under given circumstances (e.g. when the male partner is HIV infected). Couples who favoured any medically assisted reproduction technique reported that it would reduce the risk of HIV transmission much lower than timed condomless sex. Other couples reported that medically assisted methods were for those who had greater concerns of sexual transmission, especially HIV-uninfected individuals not wanting to take PrEP.

*“If they (sperms) are removed by the doctor, it is safer and at safe hands, you see…and they are inseminated to the woman and she will not get problems,…so there will be no physical contact.”* (HIV-infected male, IDI, age 44)

*“Just in case may be, the wife does not want to use that Truvada, she is seeing it as a very serious thing to take so you know, once the sperm wash is done, at least you are, you know the chances that, the infection is like zero percent, so there is no problem*. (HIV-infected males, FGD)

Multiple fertility care providers spoke about IUI using washed sperm as the ideal method and a better option than “natural” conception, due to sexual HIV transmission risk concerns. In addition, providers in fertility clinics reported conducting fertility screening procedures, such as checking the patency of the fallopian tubes and sperm count. Providers in non-fertility clinics did not discuss fertility screening and discussion of other medically assisted reproductive services focused on its high costs.

*“I will always discourage against any form of natural conception in HIV serodiscordant couples and as much as it may be possible sometimes that conception may take place without transmission this cannot be 100 percent safe so I will always want them to go the assisted way where science can help this couple achieve their fertility goal without necessarily opening them to the risk of infection*.” (Health provider, Private fertility clinic, KII)

Some providers had experiences with counselling couples who had received incorrect information from a medical provider. These providers expressed desire for safer conception guidelines since they felt that other providers were “*very challenged*” to provide these services appropriately.

*“Mmmh the challenge has been…some of them have passed through other providers and have not had best of care like there was one who was carrying the semen almost the whole morning waiting for the doctor to insert which we think is not appropriate because by the time you are ready to insert it is too late.” (*Health provider, safer conception research site, KII)

There was consensus among the providers that some medically assisted reproduction strategies, such as sperm washing, were expensive (quoted prices ranged 200–500 USD per cycle) compared to other strategies such as self-insemination or ART. Since most couples would need several cycles before successfully conceiving, these costs were perceived to be prohibitively expensive for most couples. This perspective was passed along to the couples, who reported that providers informed them about sperm washing and emphasized the high cost, discouraging them from pursuing it.

*“Mmmh, one, we thought it is a bit costly for us because we were told it is not for free. We were told it’s something like from 20,000 shillings [200USD] and above so we never even wanted to know the details*.” (HIV-infected male, IDI age 36)

*“Definitely as I said the first intervention is IUI [intrauterine insemination] which is relatively cheap and please note the use of the word relative…yeah in as much as we say one cycle success rates will be 15%. …and you don’t just do one cycle and say the IUI has failed, you understand? You do 3, you need to do minimum 3 okay, if possible 6 cycles.”* (Health provider, Fertility clinic-public and private, KII)

## Self-insemination

Many couples were aware of self-insemination, a procedure that could be done at home. However, couples reported feeling concerned about the integrity of the sperm injected and others found it “unnatural”. Additionally, some couples reported that they would prefer provider-assisted insemination. Men reported that the method of obtaining and handling semen for self-insemination was “a lot of work” but this was countered by women reporting that if the male partners wanted children, they would be agreeable to self-insemination. Providers in the public health facilities and safer conception research sites also mentioned self-insemination as a possible strategy that could be used to reduce risk of HIV transmission during the peri-conception period.

*“Eeeh as long as he wants to get a child…As long as by the way he wants a child he will agree [to self-insemination] he will agree*.” (HIV-infected females, FGD)

*“…all females…we teach them about you know artificial vaginal insemination; teach them how which is very easy and very cheap. It’s probably the cheapest intervention we have.”* (Health provider, safer conception research site, KII)

## Counselling approach

Providers reported that counselling on safer conception was individualized based on several considerations including the sex of the HIV-infected partner, ART use by the HIV-infected partner, age of the woman, the financial resources of the couple, how long they have been trying to get pregnant, and their education levels. This approach of individualized counselling was advocated by most providers irrespective of the facility in which they worked and was also favoured by couples. Additionally, some providers added that if a woman was younger, the chances of getting pregnant were higher and the women were not as hurried to conceive compared with the older women. Some couples also expressed preference for methods that did not involve direct sexual contact and would therefore only accept medically assisted reproductive techniques. Providers reported that when the man was HIV infected, it was more difficult to provide options because self-insemination was not appropriate.

*“We tailor make, when a patient walks to my clinic, I tailor make. You put all this into the option plus the cost and so they make a choice. So most probably, the people who are financially challenged, the option of ARVs and having intercourse at the time of ovulation tends to be more appealing. Those who have resources are ready to do anything. They are able to do sperm wash, they are able to do IUI, they are able to them all. So, it is really tailor made to an individual, we don’t prescribe one for everybody*.” (Health provider, Fertility clinic-public and private, KII)

The providers who worked in clinics providing safer conception services reported zero sexual and perinatal HIV transmissions, and this gave them confidence in their approach to recommend safer conception strategies. Most of the providers reported that couples conceived within six months when there was no underlying infertility issue. Therefore, in cases where couples did not achieve pregnancy in the first attempt, experience led providers to reassure the couples that there would very likely succeed in subsequent attempts.

*“I would say, actually ever since we started this…discordant couple clinic care, we have not had a seroconversion neither do we have a baby who turned out (HIV) positive*.” (Health provider, Safer conception research site, KII)

## Discussion

Health providers promoted multiple safer conception strategies for HIV serodiscordant couples. There was consensus that safer conception packages can ideally be tailored to individual/couples’ preferences to maximize use and the preventive benefit. Provider preferences and the depth of their knowledge and experiences aligned with their professional specialty; sometimes providers lacked knowledge about methods that were grounded in another specialty (i.e. fertility care providers did not fully understand the benefits of antiretrovirals while providers who were not fertility specialists less commonly discussed medically assisted reproduction). Therefore, programmes training health providers on HIV prevention and safer conception for serodiscordant couples can be cognizant of these knowledge gaps and aim to equip health providers with comprehensive knowledge of all safer conception strategies and tools to help couples overcome any stigma they may fear when seeking services.

The PrEP- and ART-experienced couples in this study were confident that these strategies were protective but some still chose to augment with timed condomless sex or self-insemination. Although a safer conception strategy that included timing condomless sex to peak fertility periods was highly acceptable to the couples, accurate knowledge about the identification of peak fertility was lacking [4,28]. Health providers desired innovative tools to support this understanding; few were familiar with existing mobile applications for tracking fertility. Studies from other countries have reported that most of the current electronic applications used to predict the fertile period have low accuracy levels, information that needs to be disseminated to couples and providers [29].

Providers’ confidence in delivering safer conception counselling was bolstered in their experiences with couples conceiving successfully without sexual or perinatal transmission of HIV. Studies conducted in diverse regions have also reported successful use of safer conception to result in conception without HIV transmission [10–12,16]. A wider sharing of these experiences and perspectives will impart recognition of safer conception and encourage providers to initiate conversation with clients about their fertility desires. However, discussion about safer conception will need to be added into already condensed time that providers have, especially in public health facilities. Additionally, providers posited that successful delivery of safer conception counselling incorporates the preferences and circumstances of each couple and customizes their safer conception package, which may overcome patient reluctance to engage health providers in safer conception counselling that has been reported in some studies [30–34]. Regardless of the gender of the HIV-positive partner, multiple strategies can be used (either singly or in combination) to minimize HIV transmission risk during pregnancy attempts. Strategies that encourage communication between health providers and couples desiring fertility will most likely result in very effective safer conception strategies for couples to consider, which could translate to higher compliance.

A strength of our study included interviewing health providers in diverse settings and talking with providers and HIV serodiscordant couples allowed us to explore specific themes across different lenses. The couples in our study were recruited from the Partners Demonstration Project therefore familiar with ART and PrEP which may limit generalizability of our findings. Future studies should be conducted among couples newly diagnosed or delaying to use antiretrovirals and in settings where medically assisted interventions are less costly. Additional studies among couples offered safer conception counselling and interventions would provide invaluable insights on the actual implementation experiences.

Based on our study findings, a basic package of safer conception strategies including ART and PrEP, education to identify peak fertility and time condomless sex, and medical history-based fertility screening, would be feasible in the Kenyan public health system. The expansion of PrEP availability and the potential for fertility services to become more widely available would provide additional tools. There is growing demand for safer conception services and providers are gaining experience and confidence providing counselling and facilitating access to interventions. Discussion between providers and couples is paramount to foster provider confidence in the delivery of services, including referral to those outside of their medical specialty, and couples’ trust in the interventions accessible to them. Should ongoing safer conception demonstration projects report success, the dissemination of lessons learned will increase awareness of safer conception services and their role to reduce sexual HIV transmission during pregnancy attempts.

## Conclusions

HIV serodiscordant couples with immediate fertility desires expressed desire for a variety of safer conception interventions, including PrEP, ART and timed condomless sex. Medically assisted reproduction was perceived to be costly but its limited availability did not allow for a comprehensive assessment of its acceptability or preferential ranking. Cross disciplinary training would equip health providers with the necessary knowledge to offer couples a comprehensive safer conception package including antiretrovirals as well as fertility care to optimize pregnancy. Safer conception counselling initiated through frequent discussions of fertility desires and integrated into HIV prevention and care for HIV serodiscordant couples offers opportunities to identify couples entering a stage of high HIV vulnerability and to encourage interventions that are available and accessible to the individual couples.
